# Assessment of central retinal thickness, choroidal thickness, and retinal nerve fiber layer in psoriasis: a spectral-domain optical coherence tomography study

**DOI:** 10.1186/s12886-021-02000-7

**Published:** 2021-05-26

**Authors:** Asena Keles Sahin, Fatma Etgü, Aslihan Uzun

**Affiliations:** 1 Department of Ophthalmology, Training and Research Hospital, Ordu University, 52000 Ordu, Turkey; 2 Department of Dermatology, Training and Research Hospital, Ordu University, Ordu, Turkey

**Keywords:** Psoriasis, Optical coherence tomography, Choroidal thickness, Macular thickness, Retinal nerve fiber layer

## Abstract

**Background:**

This study aims to evaluate choroidal thickness (CT), retinal thickness, ganglion cell-inner plexiform layer (GCIPL), and retinal nerve fiber layer (RNFL) structures in psoriasis patients using optical coherence tomography (OCT).

**Methods:**

This study included 33 psoriasis patients and 33 healthy individuals. Moreover, psoriasis patients who did not use any systemic anti-inflammatory treatment were evaluated. Retinal and choroidal images of the participants were obtained with spectral-domain OCT. Furthermore, CT was measured in the subfoveal, temporal, and nasal positions at 500-µm intervals to a distance of 1,500 μm from the foveal center.

**Results:**

The mean psoriasis area and severity index (PASI) score was 5.70 (range, 2.40–9.00). No significant differences were found in subfoveal (*p* = 0.659), temporal, and nasal CT values in psoriasis patients compared with the control group (*p* > 0.05). Similarly, no statistically significant differences were found between the groups in terms of central retinal thickness, macular GCIPL, and RNFL (*p* > 0.05). Moreover, no significant correlation exists between the duration of psoriasis disease and PASI scores and OCT parameters (*p* > 0.05).

**Conclusions:**

No significant changes in CT, ganglion cell layer, RNFL, and retinal thickness values were noted in psoriasis patients with mild to moderate mean PASI score.

## Background

Psoriasis is an immune-mediated chronic inflammatory papulosquamous skin disease that affects approximately 2 % of the population worldwide and negatively affects the quality of life [1]. Its pathogenesis is not fully known. However, genetic and environmental factors play a role. The immunopathology of psoriasis has been accepted as T cell activation, causing systemic inflammation and increased cytokine activity [2, 3]. Vascular endothelial growth factor (VEGF), hypoxia-inducible factors, and tumor necrosis factor-α (TNF-α) may be associated with disease development [4]. Moreover, psoriasis is a disease that often occurs with relapse and regression and can affect other organs other than the skin. Therefore, it can be considered a systemic disorder rather than just a skin disease [5, 6]. This disease, accompanied by systemic inflammation, is associated with multiple comorbidities (e.g. myocardial infarction and metabolic syndrome) [7]. The disease has different morphological types. This disease has been associated with ocular inflammatory diseases, particularly uveitis. Consequently, approximately 10 % of psoriasis patients have ocular findings that can affect the eyelid, conjunctiva, cornea, lens, and anterior uvea although the exact prevalence is unknown. Ocular findings usually occur bilaterally during exacerbation periods of psoriasis. Furthermore, vision-threatening complications are rare [8–11].

Optical coherence tomography (OCT) is a noninvasive method that can provide high-resolution cross-sectional images of the retina, retinal nerve fiber layer (RNFL) and choroid, and is an important tool in the diagnosis and treatment of chorioretinal diseases [12]. The choroid is one of the tissues with maximal vascularisation in the human body, which has essential roles in external retinal oxygenation and nutrition, retinal temperature regulation, and growth factor secretion [13, 14]. The enhanced depth imaging OCT (EDI-OCT) technique provides comprehensive choroidal imaging and helps in understanding the pathophysiology of ocular disorders [15–17].

Studies should be extended to the posterior part of the eye because inflammatory processes play a role in eye diseases in the anterior segment as well as in the posterior segment. Retinal and choroidal thickness (CT) has been evaluated in many systemic inflammatory diseases [18–20]. Depending on the severity of inflammatory processes, posterior ocular structures are affected in chronic inflammatory diseases. The retina and choroid tissue with extensive vascularisation are expected to be affected in chronic inflammatory diseases such as psoriasis. Studies on psoriasis patients have mostly investigated the anterior chamber, while studies evaluating the retina and choroid are fewer. Chandran et al. reported that optic nerve involvement may occur in this disease with the effect of increasing cytokines, mainly TNF-α. However, comprehensive studies evaluating RNFL thickness and macular ganglion cell-inner plexiform layer (mGCIPL) are not enough.

This study aimed to evaluate CT, retinal thickness, mGCIPL, and RNFL in psoriasis patients and compare them with healthy individuals and determine how these segments are affected by this inflammatory disease.

## Materials and methods

This cross-sectional study included 33 psoriasis patients and 33 healthy individuals. The study was conducted following the Helsinki Declaration and was approved by the Ordu University Training and Research Hospital Ethics Review Committee (No: 2020/173). Moreover, written informed consent was obtained from all participants.

### Patient Enrollment

Psoriasis patients who were followed up in Ordu University Training and Research Hospital Dermatology Clinic were included in the study. The diagnosis of psoriasis was made by dermatological and histopathological evaluations of the patients. Age- and gender-matched healthy individuals referred to the ophthalmology outpatient clinic due to minor refractive error and whose intraocular pressure (IOP) was < 21 mmHg were included as the control group. The inclusion criteria were patients > 18years, who had not used any systemic treatment for psoriasis in the last 3 months, with < 5D (spherical) and < 3D (cylindrical) refractive error, with the best-corrected visual acuity (BCVA) of 20/25 or better and with an axial length between 20 and 26 mm. The exclusion criteria were any anterior segment pathology (corneal opacities and massive cataracts that may interfere with OCT imaging), prior laser application or any ocular surgery, retinal diseases (e.g. macular degeneration), smoking history, any existing history of systemic diseases (e.g. diabetes mellitus and hypertension), glaucoma, acute or chronic uveitis, ultraviolet phototherapy, use of any ocular or systemic medication, patients whose inner and outer margin of the choroid presented poorly in OCT, pregnancy, or lactation.

### Study Protocol

All participants underwent a comprehensive ophthalmologic examination, including BCVA by using the Snellen chart, slit lamp examination, IOP measurements using a pneumotonometry (Tonoref III, Nidek Co., Ltd., Tokyo, Japan), and dilated fundus examination. Axial length was measured using A-scan ultrasonic biometry (Pac-Scan 300, Sonomed Escalon, New Hyde Park, NY, USA). Retinal, RNFL, and CT measurements following other ophthalmological examinations were performed using an SD-OCT device (Cirrus HD-OCT 4000, Carl Zeiss Meditec, Inc., Dublin, CA, USA). Moreover, ophthalmological examinations and SD-OCT measurements were performed in the morning (between 09:00 and 12:00). The right eyes of all participants were evaluated in the measurements. Furthermore, ophthalmological examinations of all the patients were performed by the same researcher.

Psoriasis patients used topical medications only for the last 3 months without any systemic anti-inflammatory treatment. The evaluation of psoriasis patients was performed by the same dermatologist. Disease severity was evaluated through the psoriasis area and severity index (PASI) score. Psoriasis was considered mild to moderate and severe if the PASI scores were < 10 and ≥ 10, respectively. The arthritis history of the patients was recorded.

**Fig. 1:**
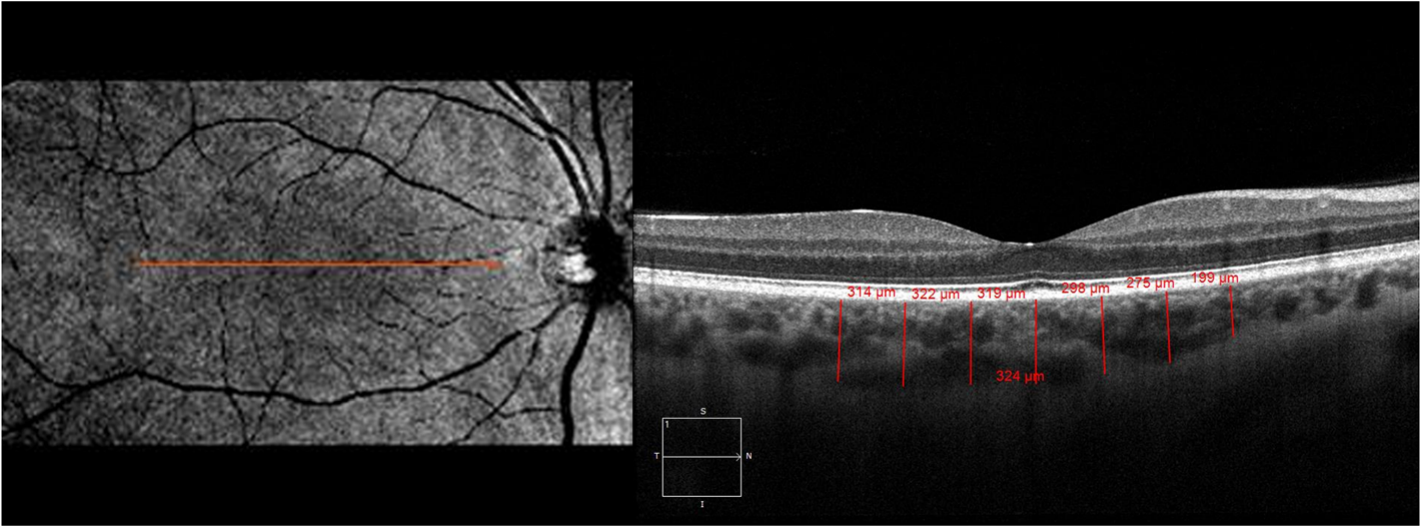
Enhanced depth optical coherence tomography image of choroidal thickness in a psoriasis patient

**Fig. 2:**
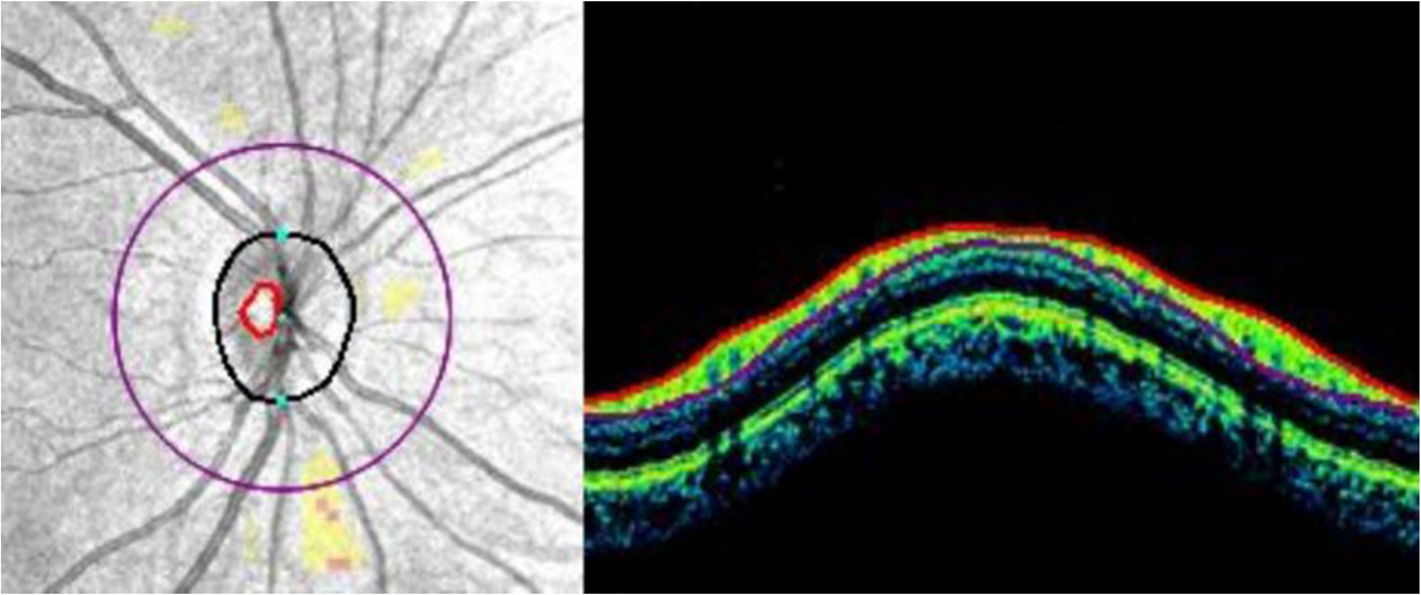
Optical coherence tomography image showing peripapillary retinal nerve fiber layer in a psoriasis patient

All OCT scans and measurements were obtained by the same experienced technician through pupil dilation (1 % tropicamide and 2.5 % drops phenylephrine). Those scans of the macula and optic nerve head with a signal strength of > 7/10 were used for analysis. Central retinal thickness (the central 1-mm diameter circle) and average mGCIPL scans were performed automatically using a macular cube 512 × 128 scan protocol (128 consecutive line scans). This protocol has a scan area of 6 × 6 mm of the retina and macular thickness is calculated in microns in an area correspondent to the Early Treatment Diabetic Retinopathy Study grid. The average GCIPL thickness was measured in an elliptical annulus with a 2.0 mm vertical and 2.4 mm horizontal radius, excluding a central elliptical area (0.5 mm vertical and 0.6 mm horizontal radius). The choroidal images were obtained using the HD Line Raster (EDI) protocol. The central fovea in the horizontal section image passing through the fovea and the nasal (three points) and temporal (three points) segments at 500-µm intervals to a distance of 1,500 μm from the fovea were used as measurement points for CT. The CT was measured manually from the outer part of the hyperreflective line corresponding to the retinal pigment epithelium, perpendicular to the inner surface of the sclera (Fig. 1). Consequently, CT measurements were performed by two experienced ophthalmologists (AKS and AU) who were masked in different sessions using manual calipers of Cirrus HD-OCT software, and the measurements were averaged for analysis. Peripapillary RNFL thickness was measured with an Optic Disc Cube 200 × 200 protocol along a circle of 1.73-mm radius around the optic disc (Fig. 2). For peripapillary RNFL thickness, the mean (360°) and quadrants (superior, nasal, inferior, and temporal) values determined automatically by the device software were recorded.

### Statistical analysis

The sample size is based on the literature of the difference observed in CT in psoriasis patients. A power of 80 % and a confidence level of 95 % yielded the sample size. All data were analyzed using the SPSS statistical software package, version 21.0 (SPSS Inc., Chicago, IL, USA). Categorical variables were expressed as frequency and percent and were compared using the chi-square test. A normality check was performed using the Kolmogorov–Smirnov test. Normally distributed data were expressed as mean and standard deviation and were compared using an independent *t*-test. Non-normally distributed data were presented as median (first to third quartiles) and were compared using the Mann–Whitney test. OCT values were compared with the duration of psoriasis and PASI values using the Spearman correlation coefficient. *P* < 0.05 was accepted as a statistically significant level.

## Results

The demographic and clinical characteristics of the participants in the study groups are presented in Table 1. No significant differences were observed between the groups in terms of gender distribution, age, visual acuity, spherical equivalent, IOP, and axial length values (*p* > 0.05). Myopic refraction was present in 17 (48.48 %) patients in the psoriasis group and 20 (60.61 %) patients in the control group. None of the participants had a spherical equivalent higher than − 2 or + 2 diopters. None of the patients had posterior or anterior uveitis. Similarly, no pathology was found in any of the patients in the posterior segment examinations. Although no joint involvement exists in the control examination of the patients, 12 (36.36 %) had a history of previous arthritis.

**Table 1:** Demographic and clinical characteristics of the patients

Parameter	Psoriasis (*n* = 33)	Control (*n* = 33)	*P* value
Age (years)	31 (23–45)	36 (27–40)	0.959^a^
Gender (Male/Female)	16/17	16/17	1.000^b^
Visual acuity (Snellen chart)	0.99 ± 0.04	0.98 ± 0.06	0.990^c^
Spherical equivalent (D) (mean ± SD)	−0.13 ± 0.71	−0.50 ± 0.89	0.263^c^
IOP (mmHg)	16 (14–19)	16 (14–18)	0.538^a^
Axial length (mm) (mean ± SD)	22.68 ± 0.65	22.89 ± 0.72	0.213^c^

*IOP* intraocular pressure, *D* diopter, *SD* standard deviation

^a^Mann–Whitney *U* test, ^b^chi-square test, ^c^independent *t*-test

The mean duration of psoriasis of the patients in the psoriasis group was 4 years (range, 2–10 years), and the mean PASI score was 5.70 (range, 2.40–9.00). Eight (24.24 %) patients had a severe disease score when the patients were evaluated according to the disease severity.

The OCT parameters of the groups are shown in Table 2. Subfoveal (*p* = 0.659), temporal and nasal CT values were insignificantly higher in psoriasis patients (temporal and nasal positions at 500-µm intervals from the fovea, *p* = 0.792, *p* = 0.874, *p* = 0.609, *p* = 0.656, *p* = 0.662 and *p* = 0.750, respectively). No statistically significant differences were found between the groups regarding retinal thickness, mGCIPL, and average and quadrants RNFL values (*p* > 0.05).

**Table 2:** Optical coherence tomography parameters among the groups

Parameter (µm)	Psoriasis (*n* = 33)	Control (*n* = 33)	*P* value
CRT	253.79 ± 20.77	254.33 ± 19.51	0.913^b^
Subfoveal CT	360.97 ± 67.10	354.15 ± 57.63	0.659^b^
Temporal CT			
500 μm	345.06 ± 63.29	341.21 ± 54.26	0.792^b^
1000 μm	335.18 ± 62.63	332.91 ± 53.40	0.874^b^
1500 μm	328.61 ± 62.57	321.00 ± 57.68	0.609^b^
Nasal CT			
500 μm	336.21 ± 63.37	329.76 ± 53.56	0.656^b^
1000 μm	318.52 ± 67.46	311.85 ± 55.13	0.662^b^
1500 μm	304.85 ± 66.45	299.97 ± 57.22	0.750^b^
Average RNFL	94.79 ± 13.50	94.76 ± 7.60	0.991^b^
Temporal RNFL	66.76 ± 12.48	66.55 ± 6.63	0.932^b^
Nasal RNFL	71.00 ± 11.07	68.82 ± 9.88	0.401^b^
Superior RNFL	119.12 ± 20.20	117.55 ± 13.86	0.713^b^
Inferior RNFL	126 (116–130)	125 (121–134)	0.480^a^
Average mGCIPL	86 (83–89)	87 (84–88)	0.964^a^

Data are given as mean ± standard deviation or median (minimum–maximum) according to normality of distribution. *CRT* central retinal thickness, *CT* choroidal thickness, *RNFL* retinal nerve fiber layer, *mGCIPL* macular ganglion cell-inner plexiform layer

^a^Mann–Whitney *U* test, ^b^independent *t*-test

No significant correlation was found between the duration of psoriasis disease and PASI scores and OCT parameters (Table 3).

**Table 3:** Correlation analysis between psoriasis characteristics and OCT parameters

		PASI	Duration of psoriasis
CRT	*r*	−0.162	0.024
	p*	0.369	0.894
Subfoveal CT	*r*	0.066	0.024
	p*	0.716	0.894
Temporal CT 500 μm	*r*	0.090	0.158
	p*	0.620	0.381
1000 μm	*r*	0.047	0.121
	p*	0.795	0.501
1500 μm	*r*	0.045	0.227
	p*	0.803	0.204
Nasal CT 500 μm	*r*	0.084	−0.022
	p*	0.643	0.904
1000 μm	*r*	0.105	0.103
	p*	0.562	0.569
1500 μm	*r*	0.125	0.146
	p*	0.487	0.416
Average RNFL	*r*	−0.223	−0.332
	p*	0.213	0.059
Temporal RNFL	*r*	0.027	−0.273
	p*	0.881	0.124
Nasal RNFL	*r*	−0.211	0.056
	p*	0.237	0.758
Superior RNFL	*r*	−0.270	−0.330
	p*	0.129	0.061
Inferior RNFL	*r*	−0.202	−0.317
	p*	0.259	0.073
Average mGCIPL	*r*	−0.174	−0.190
	p*	0.332	0.289

*OCT* optical coherence tomography, *CRT* central retinal thickness, *CT* choroidal thickness, *RNFL* retinal nerve fiber layer, *mGCIPL* macular ganglion cell-inner plexiform layer

*Spearman correlation analysis

## Discussion

Systemic involvement and ocular surface affection in psoriasis have been shown in various studies. However, insufficient data about retina, choroid, and retinal nerve fiber exist. We investigated choroid and retinal structures and their relationship with the severity and duration of the disease in psoriasis. In the current study, we reported that CT was insignificantly thicker in psoriasis patients than in the control group.

Ocular involvement in psoriasis may directly develop with skin lesions due to an immune-mediated inflammatory process or as a complication due to treatments. Blepharitis, conjunctivitis, and keratoconjunctivitis sicca have been frequently reported as anterior segment involvement [9]. Uveitis is mostly seen as anterior uveitis in this disease, and it is thought to occur more frequently in severe psoriasis and psoriatic arthritis [21]. In this study, 12 patients had a history of arthritis. However, no evidence of anterior or posterior uveitis was found in any patient.

TNF-α is an important proinflammatory cytokine that plays a role in the pathogenesis of psoriasis disease. A direct correlation exists between disease severity and TNF concentrations [22]. Moreover, TNF-α was found to play a role in the pathogenesis of inflammatory and neovascular disorders in the eye [23]. The in vivo retinal injury model showed that TNF-α plays a largely deleterious role in ischemia–reperfusion injury, and retinal function is partially protected by direct neutralization of this cytokine [24]. Furthermore, Derevjanik et al. [25] reported that the injection of TNF-α into the eyes of the animals disrupts the blood–retinal barrier. The successful use of anti-TNF agents in the ocular involvement of inflammatory diseases supports this molecule’s role in ocular involvement in inflammatory diseases [23]. Thus, these data support the place of TNF-α in the pathogenesis of ocular findings due to psoriasis. Moreover, an experimental study showed that inflammatory cytokines increase VEGF secretion, which is a crucial element for pathological ocular neovascularisation, from human retinal pigment epithelial cells and choroidal fibroblasts [26]. Consequently, choroidal tissue with dense vascularisation is affected in various systemic inflammatory diseases, and posterior segment structures have been the subject of psoriasis research.

The choroid is thicker in patients with severe psoriasis. Furthermore, Ersan et al. [27] evaluated psoriasis patients according to the PASI score and showed that subfoveal CT was significantly thicker in patients with severe psoriasis compared with mild psoriasis and the control group. This affection of the choroid was thought to be a result of the inflammatory cascade involved in the disease pathogenesis. Aksoy et al. [28] reported the CT was significantly thicker in the subfoveal, nasal, and temporal segments 500 μm away from the fovea compared with the mild and control groups in patients with severe psoriasis. In the comparison made between the mild and control groups, no significant difference was found in CT. Different investigators reported no significant correlation between disease duration and CT. Moreover, Türkcü et al. [29] found that CT is significantly higher in psoriasis, and no correlation with PASI score and disease duration was noted. Most of the patients (75.76 %) in this study had mild to moderate psoriasis. Similar to the reported results, CT was found to be thicker in psoriasis patients than in the control group, but this thickness was not statistically significant. Thus, the lower number of patients with severe disease score (24.24 %) may be effective in this insignificant increase in thickness. Additionally, no significant change was found in retinal thickness. Consequently, no correlation was found between retinal and CT as well as disease duration and disease activity score.

As an extension of the central nervous system, the inner layers of the retina may indicate neurodegenerative processes in the brain [30]. Abnormalities in RNFL and ganglion cell layer have been reported with OCT studies in Alzheimer’s disease, Parkinson’s disease, and multiple sclerosis [31–33]. TNF-a, which has an essential effect on the inflammatory process in psoriasis, may be involved in the pathogenesis of neurodegenerative diseases [34]. Moreover, Perossini et al. [35] reported that abnormal visual-evoked potential parameters in psoriasis patients, which may be a sign of optic neuritis, were noted. This situation may be caused axonal damage due to autoimmune activity caused by the increase of TNF-a and other cytokines in the blood. On the one hand, Kitaoka et al. [36] demonstrated axonal degeneration and retinal ganglion cell loss with intravitreal TNF-alpha injection in rats. On the other hand, Aksoy et al. [28] reported a significant decrease in RNFL in patients with severe psoriasis. This study found no significant changes in RNFL and ganglion cell layer thicknesses in psoriasis patients compared with the control group.

Our study has some limitations. First, our sample size was limited and did not include participants from different ethnicities and different geographical regions. The second limitation was that it could not be analyzed as a separate group due to the low number of patients with severe disease scores. Third, The CT was measured manually using calipers of the OCT device. Additionally, a standard protocol for systematical identification of glaucoma patients was not provided. Using pneumotonometry instead of Goldmann applanation tonometer for IOP measurements and determining the IOP cut-off value as 21 mmHg may be important when interpreting the results of the study. These points may limit the validity of our study results to elderly patients. Furthermore, the average PASI score was relatively low. The severe disease is considered an indicator of an intense inflammatory process, which may be the reason for the lack of significant changes in CT values and the absence of significant differences in other OCT parameters.

In conclusion, choroids were not significantly thicker in psoriasis patients. Consequently, no significant changes were noted in the values of the ganglion cell layer, RNFL, and retinal thickness. Determining retinal structures and changes in the choroid in psoriasis, which is a systemic inflammatory disease, will be useful in elucidating the pathophysiology of ocular effects and diagnosing complications. Furthermore, changes in CT can provide information about the inflammatory activity of the disease. Thus, studies to be conducted with larger patient groups will help understand the ocular effects of this disease.

## Data Availability

The datasets generated and/or analyzed during the current study are not publicly available due to local data protection laws but are available from the corresponding author on reasonable request.
